# Manganese Alkyl Carbonyl Complexes: From Iconic Stoichiometric Textbook Reactions to Catalytic Applications

**DOI:** 10.1021/acs.accounts.2c00470

**Published:** 2022-09-08

**Authors:** Stefan Weber, Karl Kirchner

**Affiliations:** Institute of Applied Synthetic Chemistry, Technical University Vienna, Getreidemarkt 9, A-1060 Vienna, Austria

## Abstract

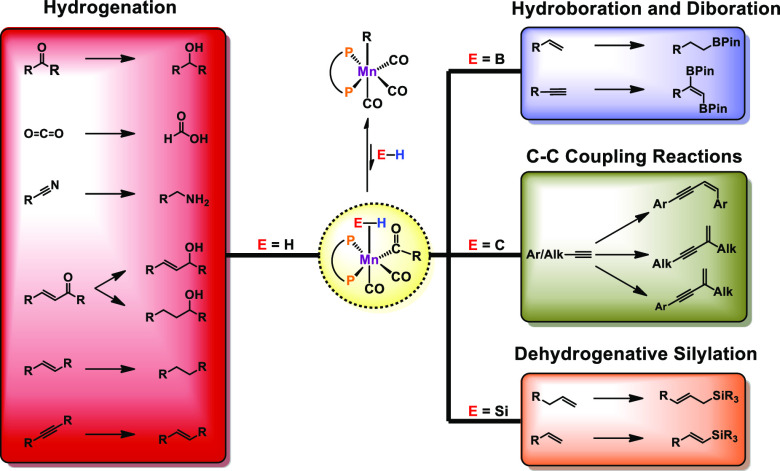

The activation of weakly polarized bonds represents a challenging, yet highly valuable process. In this context, precious metal catalysts have been used as reliable compounds for the activation of rather inert bonds for the last several decades. Nevertheless, base-metal complexes including cobalt, iron, or nickel are currently promising candidates for the substitution of noble metals in order to develop more sustainable processes. In the past few years, manganese(I)-based complexes were heavily employed as efficient catalysts for (de)hydrogenation reactions. However, the vast majority of these complexes operate via a metal–ligand bifunctionality as already well implemented for precious metals decades ago. Although high reactivity can be achieved in various reactions, this concept is often not applicable to certain transformations due to outer-sphere mechanisms. In this Account, we outline the potential of alkylated Mn(I)-carbonyl complexes for the activation of nonpolar and moderately polar E–H (E = H, B, C, Si) bonds and disclose our successful approach for the utilization of complexes in the field of homogeneous catalysis. This involves the rational design of manganese complexes for hydrogenation reactions involving ketones, nitriles, carbon dioxide, and alkynes. In addition to that, the reduction of alkenes by dihydrogen could be achieved by a series of well-defined manganese complexes which was not possible before. Furthermore, we elucidate the potential of our Mn-based catalysts in the field of hydrofunctionalization reactions for carbon–carbon multiple bonds. Our investigations unveiled novel insights into reaction pathways of dehydrogenative silylation of alkenes and *trans*-1,2-diboration of terminal alkynes, which was not yet reported for transition metals. Due to rational catalyst design, these transformations can be achieved under mild reaction conditions. Delightfully, all of the employed complexes are bench-stable compounds. We took advantage of the fact that Mn(I) alkyl complexes are known to undergo migratory insertion of the alkyl group into the CO ligand, yielding an unsaturated acyl intermediate. Hydrogen atom abstraction by the acyl ligand then paves the way to an active species for a variety of catalytic transformations which all proceed via an inner-sphere process. Although these textbook reactions have been well-known for decades, the application in catalytic transformations is still in its infancy. A brief historical overview of alkylated manganese(I)–carbonyl complexes is provided, covering the synthesis and especially iconic stoichiometric transformations, e.g., carbonylation, as intensively examined by Calderazzo, Moss, and others. An outline of potential future applications of defined alkyl manganese complexes will be given, which may inspire researchers for the development of novel (base-)metal catalysts.

## Key References

- WeberS.; StögerB.; VeirosL. F.; KirchnerK.Rethinking Basic Concepts - Hydrogenation of Alkenes Catalyzed by Bench-Stable Alkyl Mn(I) Complexes. ACS Catal.2019, 9, 9715–9720.^1^*A variety of bisphosphine-supported manganese alkyl carbonyl complexes were synthesized and applied for additive-free hydrogenation of alkenes. Upon rational design, hydrogenation of mono- and 1,1-disubtitued alkenes could be achieved at room temperature*.
- WeberS.; ZobernigD.; StögerB.; VeirosL. F.; KirchnerK.Efficient hydroboration of alkenes and *trans*-diboration of alkynes catalyzed by Mn(I) alkyl complexes. Angew. Chem., Int. Ed.2021, 60, 24488–2449210.1002/anie.202110736PMC859682534435424.^2^*Efficient and selective anti-Markovnikow hydroboration of terminal alkenes by a manganese alkyl carbonyl complex was reported. Furthermore, fully acceptorless trans-1,2-diboration of terminal alkynes, including mechanistic investigations, was presented*.
- WeberS.; GlavicM.; StögerB.; PittenauerE.; PodewitzM.; VeirosL. F.; KirchnerK.Manganese-Catalyzed Dehydrogenative Silylation of Alkenes Following two Parallel Inner-Sphere Pathways. J. Am. Chem. Soc.2021, 143, 17825–178323464406410.1021/jacs.1c09175PMC8554758.^3^*Highly E-selective dehydrogenative silylation of monosubstituted alkenes at mild reaction condition was disclosed. Mechanistic investigations revealed the presence of two parallel pathways—one requiring an alkene substrate as the sacrificial agent and one being acceptorless involving dihydrogen formation*.
- WeberS.; VeirosL. F.; KirchnerK.Selective Manganese-Catalyzed Dimerization and Cross Coupling of Terminal Alkynes. ACS Catal.2021, 11, 6474–64833412348410.1021/acscatal.1c01137PMC8185884.^4^*A rare example of manganese-catalyzed dimerization and cross-coupling of alkynes was described. Interestingly, aryl-based alkynes gave Z-1,3-enynes, whereas dimerization or cross-coupling of aliphatic substrates provided the gem-1,3-enyne products*.

## Introduction

**Scheme 1:**
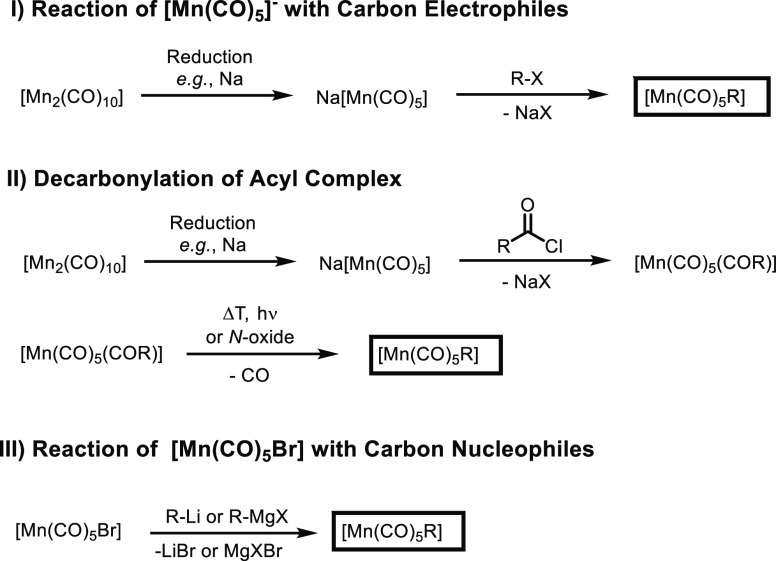
Synthesis Routes toward Manganese Alkyl and Aryl Carbonyl Complexes

Transition metal alkyl complexes represent a unique compound class in modern organometallic chemistry.^5^ Alkyl carbonyl complexes constitute an interesting subclass, since these compounds can be utilized as model compounds for applications such as the Monsanto acetic acid process,^6^ hydroformylation reactions,^7^ or Fischer–Tropsch synthesis.^8^ In this context, manganese alkyl carbonyl complexes represent the first examples of alkyl carbonyl complexes, being synthesized in 1957 by Coffield and co-workers.^9^ In the original synthesis, [Mn_2_(CO)_10_] is reduced with Na/Hg or dispersed sodium to give the nucleophilic complex Na[Mn(CO)_5_], which is subsequently treated with the electrophilic alkylation agent methyl iodide or dimethyl sulfate to yield pure [Mn(CO)_5_(CH_3_)] (Scheme 1). This synthetic route was shown to be rather general for the synthesis of a variety of different alkyl-based complexes [Mn(CO)_5_R]^10^ and tolerates neutral coligands such as 2,2′-bipyridine (bipy) in *fac*-[Mn(bipy)(CO)_3_R].^11^

An alternative route represents the decarbonylation of manganese acyl carbonyl complexes [Mn(CO)_5_(COR)]. In this procedure, the Na[Mn(CO)_5_] anion is reacted with acid chlorides, giving rise to [Mn(CO)_5_(COR)]. Decarbonylation of these acyl complexes at elevated reaction temperatures yields manganese alkyl carbonyl complexes upon carbon monoxide release.^12^ Notably, the reaction rate of decarbonylation can be drastically increased by addition of trimethylamine *N*-oxide or upon irradiation.^13^ This procedure provides synthetic access to manganese aryl carbonyl complexes, which cannot be synthesized by the reaction of aryl halides with Na[Mn(CO)_5_].

Furthermore, the reaction of nucleophilic alkylation agents, such as organolithium or Grignard reagents, with the electrophilic manganese center in [Mn(CO)_5_Br] displays an additional option for the synthesis of manganese alkyl/aryl carbonyl complexes. In fact, [Mn(CO)_5_Ph]^14^ and the benzyl substituted congener [Mn(CO)_5_(CH_2_Ph)]^15^ were successfully synthesized via this route employing phenyl lithium and benzyl magnesium chloride, respectively. However, low yields are attributed to these synthetic approaches due to the formation of [Mn_2_(CO)_10_] as a result of a single electron transfer reaction as well as other side reactions.

## Stoichiometric Reactions

**Scheme 2:**
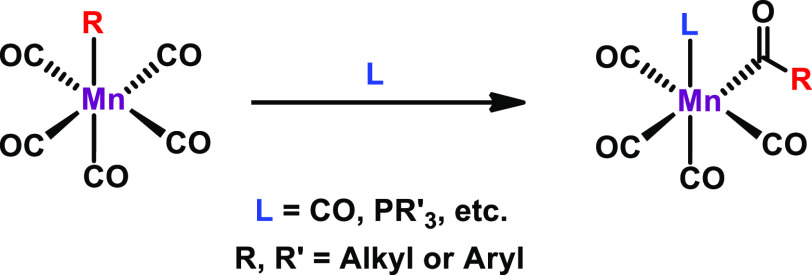
Ligand-Induced Migratory Insertion of an Alkyl or Aryl Group in the Adjacent CO Ligand

Due to their high stability and convenient synthesis, manganese alkyl and aryl carbonyl complexes were intensively investigated over the last decades.^16^ Among all investigated transformations, ligand-induced migratory insertion of the alkyl/aryl ligand into the carbonyl motif, or vice versa, has been explored most intensively. A general reaction pattern of migratory insertion of the alkyl/aryl ligand into the CO ligand upon coordination of an entering neutral ligand is depicted in Scheme 2.

In this context, the reaction of manganese alkyl and aryl carbonyl complexes with carbon monoxide constitutes the first exhaustively studied migratory insertion reaction.^12^ This well-known textbook reaction was the subject of manifold mechanistic investigations. Calderazzo and co-workers investigated whether the alkyl group is inserted into the CO ligand or if the carbonyl ligand is migrating. In fact, the researchers determined that the alkyl group and not the CO is migrating upon employment of ^13^C-enriched carbon monoxide.^17^ In another seminal contribution, Calderazzo and Cotton determined the activation energy for the carbonylation of [Mn(CO)_5_(CH_3_)].^18^ Since the value of 14.8 kcal/mol is far below the reported dissociation energy of the Mn–C bond (44 kcal/mol),^19^ a concerted reaction mechanism was proposed.

Based on these fundamental findings, two pathways for the carbonylation of [Mn(CO)_5_(CH_3_)] without solvent mediation were suggested based on both experimental^20^ and computational investigations.^21^ One route includes the formation of an η^2^-acyl intermediate; the other pathway postulates an intermediate which is stabilized by a C–H agostic acyl species. Furthermore, a solvent-mediated mechanism including coordination of the solvent to the coordinatively unsaturated acyl complex was proposed.^22^ It should be noted that carbonylation rates of [Mn(CO)_5_(CH_3_)] are drastically increased in polar solvents featuring electron-donating properties.^23^

**Scheme 3:**
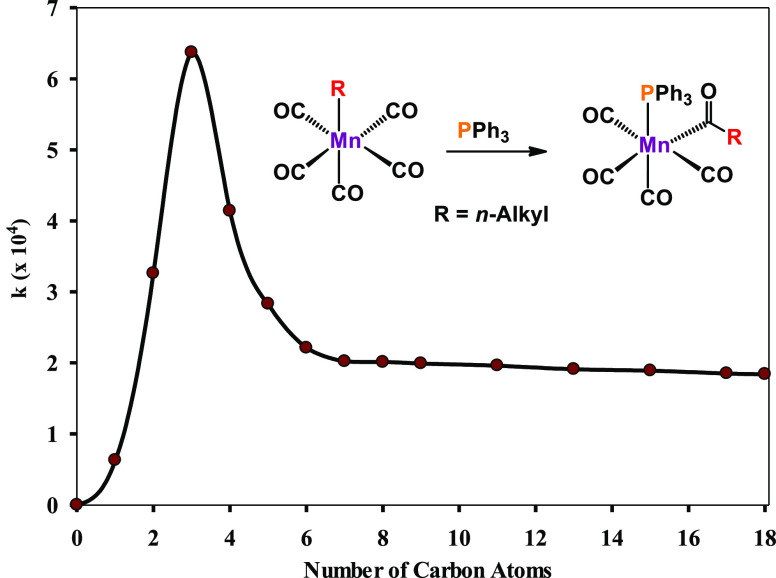
Reaction of Manganese Carbonyl Alkyl Complexes [Mn(CO)_5_R] (R = *n*-Alkyl Groups) with Triphenylphosphine

Interestingly, the migratory aptitude in carbonylation reactions depends on the nature of the alkyl ligand in [Mn(CO)_5_R]. Kinetic experiments revealed the following trend for the rate of carbonylation: *n*-Pr > Et > CH_2_C_6_H_5_ > Ph > Me ≫ CF_3_.^24^ In this context, the nucleophilicity of the alkyl/aryl group seems to play a dominant role. A similar trend was found by Moss and co-workers for the reaction of [Mn(CO)_5_R] (R = *n*-alkyl) with triphenylphosphine as the entering ligand as depicted in Scheme 3.^25^ Surprisingly, the reaction rate decreased from *n*-propyl to *n*-heptyl. With even longer chain lengths, no significant changes in reactivity were observed. These findings were attributed to the fact that for R = CH_3_ to *n*-C_3_H_7_ the R group becomes increasingly electron donating in nature which results in rate acceleration. However, when R becomes larger than *n-*propyl, the electronic effect is more or less constant and steric effects start to take over, resulting in rate retardation.

## Hydrogenation of Polarized Multiple Bonds

**Scheme 4:**
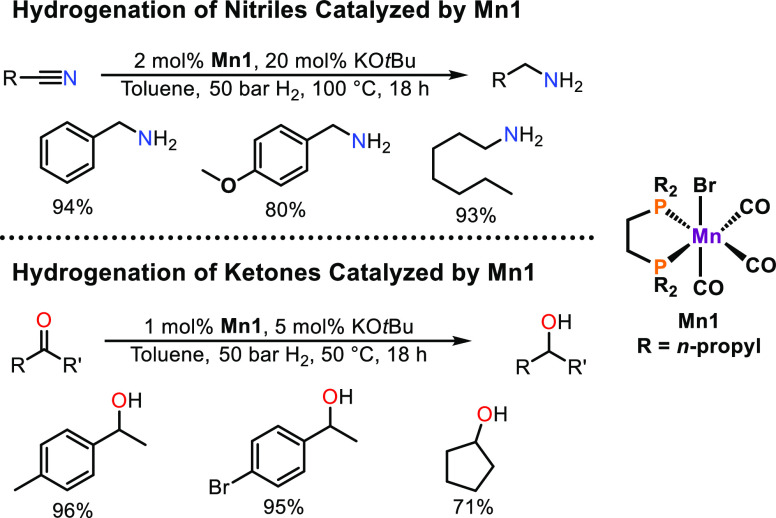
Hydrogenation of Nitriles and Ketones Catalyzed by **Mn1**

In the past few years, manganese carbonyl complexes were employed as efficient catalysts for the hydrogenation of (polarized multiple) bonds.^26^ Thus far, the vast majority of such complexes operate via a metal–ligand bifunctionality (MLB),^27^ resulting in outer-sphere reaction modes. In contradiction to MLB-based reactivity, our group decided to explore the potential of bisphosphine-supported manganese(I) complexes which are *not* capable of MLB (Scheme 4). Delightfully, **Mn1** was found to be active for the hydrogenation of nitriles in the presence of KO*t*Bu as base at elevated temperatures.^28^ A broad variety of different aromatic and aliphatic nitriles, including dinitriles, were smoothly reduced to the corresponding amines. Interestingly, other functional groups such as esters and alkynes were completely unaltered, whereas conjugated C=C bonds were only reduced to a small extend. Furthermore, **Mn1** is capable of the reduction of ketones under milder reaction conditions and a reduced amount of base.

**Scheme 5:**
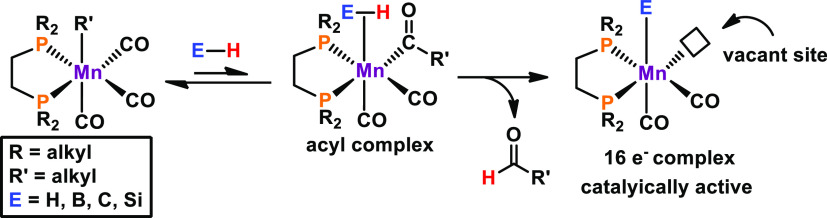
Catalyst Design and E–H Bond Activation by Manganese Carbonyl Alkyl Complexes

Preliminary mechanistic studies focused on the detected tricarbonyl hydride complex in the reaction mixture, and we proposed an outer sphere mechanism. However, later considerations involved the formation of an alkoxide-coordinated manganese tricarbonyl complex, which is able to undergo migratory insertion of the alkoxide ligand into the neighboring carbonyl moiety. If the bromide ligand is replaced by an alkyl ligand, entering ligands, containing an E–H (E = H, B, C, Si) bond, can facilitate migratory insertion into an adjacent CO ligand, giving rise to an acyl complex (Scheme 5). The basic acyl moiety may abstract the hydrogen atom from the incoming ligand, yielding a 16e^–^ complex upon release of the aldehyde. The substrate can bind to the catalytically active unsaturated complexes, resulting in an *inner-sphere* reaction pathway. In fact, E–H bonds in which the hydrogen atom possesses protic or hydridic character or is unpolarized (as in H_2_) can be activated via hydrogen atom abstraction by the acyl ligand.

**Scheme 6:**
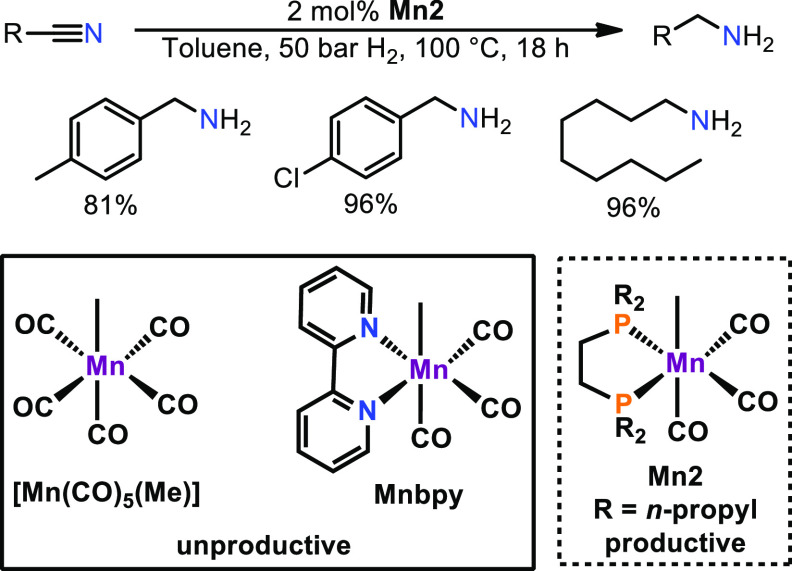
Additive-Free Hydrogenation of Nitriles Catalyzed by **Mn2**

As a proof of concept, the bromide ligand in **Mn1** was substituted by a methyl group, yielding **Mn2**, and employed for the hydrogenation of nitriles (Scheme 6).^29^ Pleasantly, **Mn2** gave similar results to **Mn1**. Nevertheless, a reaction temperature of 100 °C is required for this transformation. However, in contrast to its bromide congener, **Mn2** is able to hydrogenate nitriles in an additive-free manner. It should be noted that neither the well-known compound [Mn(CO)_5_(CH_3_)] nor the bipy complex *fac*-[Mn(bipy)(CO)_3_(CH_3_)] (**Mnbipy**) showed product formation in the hydrogenation of nitriles. The strong donor properties in combination with the increased steric demand of the bisphosphine ligand seem to be vital for the reactivity in hydrogenation reactions. Mechanistic consideration gave rise to a multifaceted reaction mechanism. **Mn2** is activated upon migratory insertion of the methyl group into the carbon monoxide ligand due to H_2_ coordination. The strongly basic acyl ligand splits dihydrogen, resulting in the formation of a hydride complex containing a weakly bonded aldehyde ligand. Coordination of the nitrile substrate followed by stepwise reduction over various intermediates then yields the amine product.

**Scheme 7:**
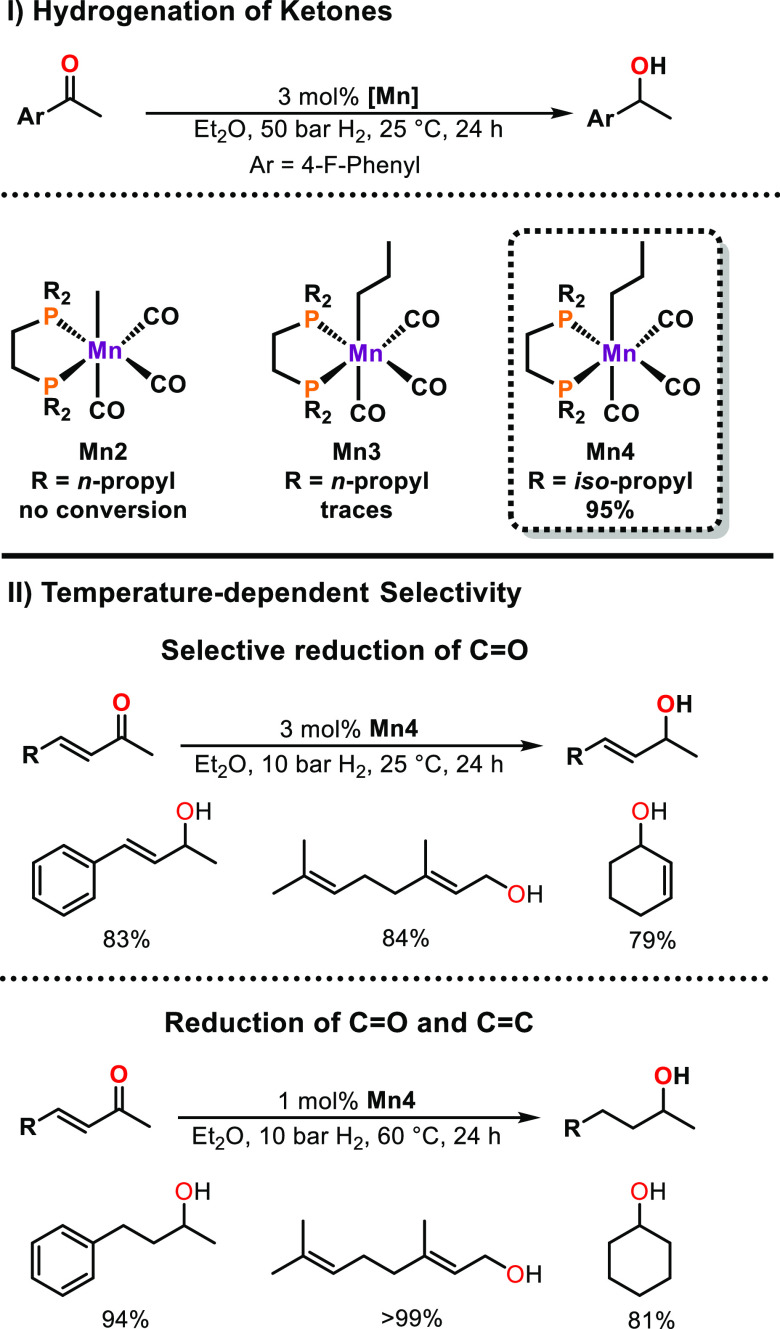
Hydrogenation of α,β-Unsaturated Ketones Catalyzed by **Mn4**

Based on our results on base-free nitrile reduction and the pioneer contributions of Calderazzo, Moss, and others, the role of steric parameters for the hydrogenation of ketones was investigated. For this purpose, the steric demand of the bisphosphine ligand as well as the chain length of the alkyl ligand was altered (Scheme 7).^30^ In fact, high reactivity was only found for the sterically more demanding bis(diisopropylphosphino)ethane (DIPPE) ligand in combination with a *n*-propyl group as anionic ligand at room temperature.

**Scheme 8:**
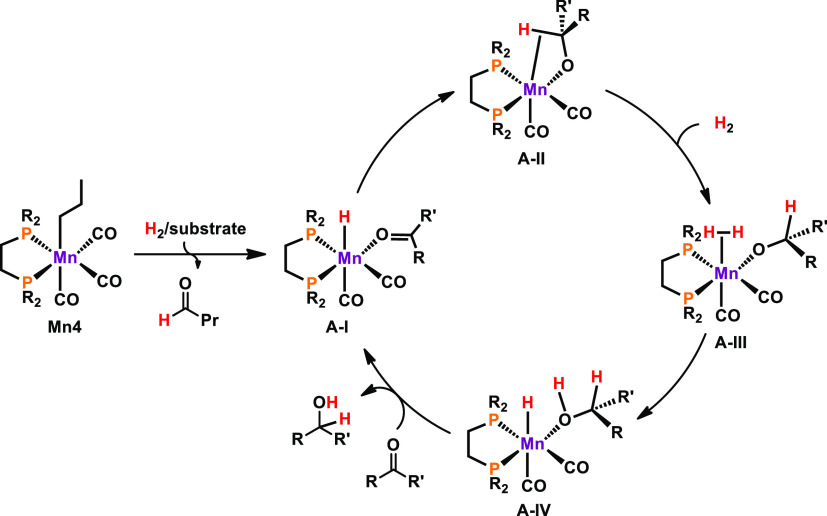
Simplified Reaction Mechanism for the Reduction of Ketones

Interestingly, decreasing the pressure from 50 to 10 bar resulted in an increase in reactivity. Remarkably, **Mn4** shows high chemoselectivity for the reduction of the carbonyl group in α,β-unsaturated ketones and aldehydes. However, if the reaction temperature is increased to 60 °C, the conjugated C=C moiety is reduced as well. This type of temperature-dependent selectivity may be of interest in various synthetic applications in organic chemistry. Furthermore, the reaction mechanism was studied by means of DFT calculations. In contrast to the vast majority of manganese-based catalysts, **Mn4** operates via an inner-sphere mechanism. A simplified reaction mechanism is presented in Scheme 8.

In the first step, **Mn4** is activated by migratory insertion of the alkyl group into the adjacent CO ligand upon coordination of dihydrogen. Heterolytic cleavage of ligated H_2_ by the acyl ligand and substitution of loosely bonded *n*-butanal by the ketone substrate results in the formation of hydride species **A-I**. Rearrangement of the O-coordinated ketone to the side-on η^2^-ligated substrate allows a nucleophilic attack of the hydride ligand on the electrophilic carbon, giving rise to **A-II**, being stabilized by an agostic C–H interaction. Coordination of dihydrogen yields κ^1^-*O* bonded complex **A-III**. Cleavage of the H–H bond by the alkoxide ligand results in the formation of hydride species **A-IV**. The catalytic cycle is closed upon ligand substitution of alcohol product by ketone substrate.

**Scheme 9:**
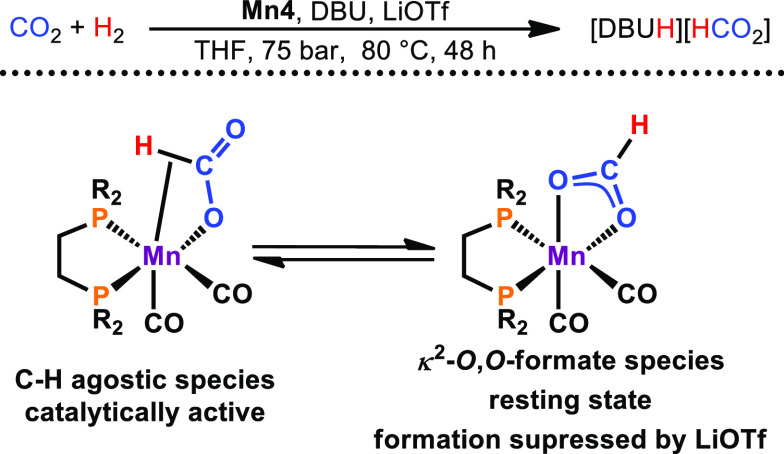
Lewis Acid Assisted Hydrogenation of Carbon Dioxide to Formate Catalyzed by **Mn4**

In cooperation with the Gonsalvi group, the potential of manganese alkyl carbonyl complexes in the hydrogenation of carbon dioxide was explored. In fact, **Mn4** was found to be capable of converting CO_2_ to formate with turnover numbers (TONs) of almost 2000 in the presence of base (Scheme 9).^31^ Furthermore, the addition of catalytic amounts of lithium triflate as the Lewis acid was found to be crucial to achieve high reactivity. This is attributed to the prevention of the formation of κ^2^-*O*,*O*-formate species as off-cycle species.

## Hydrogenation of Unpolarized Multiple Bonds

**Scheme 10:**
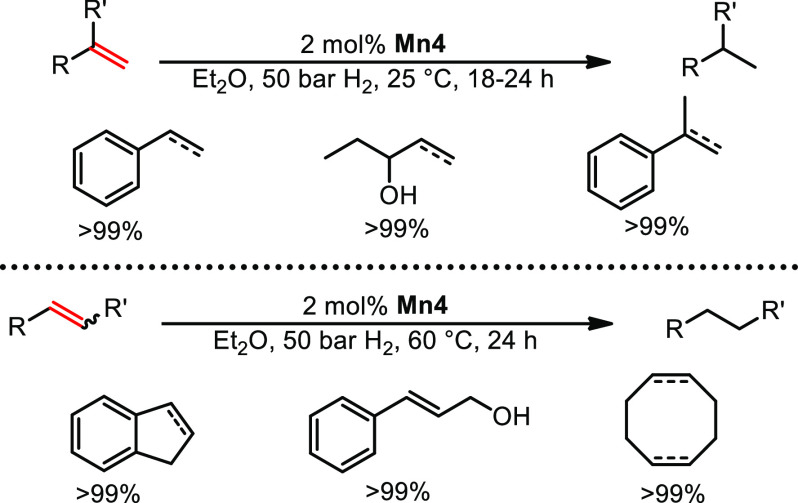
Hydrogenation of Mono- and Disubstituted Alkenes Catalyzed by **Mn4**

Apart from the hydrogenation of polarized multiple bonds, the reduction of alkenes or alkynes was achieved by manganese alkyl carbonyl complexes. Upon systematically altering the steric demand of the bisphosphine ligand and the chain length of the alkyl ligand, hydrogenation of mono- and 1,1-disubstituted alkenes could be achieved at room temperature by **Mn4**.^1^ Reduction of 1,2-disubstituted alkenes required a reaction temperature of 60 °C (Scheme 10). A broad array of functional groups including halides, amines, esters, and anhydrides was left unaltered under the employed reaction conditions. It should be noted that the complexes [Mn(CO)_5_(CH_3_)], Mnbipy, and *fac*-[Mn(DIPPE)(CO)_3_H] did not show any catalytic activity. Additionally, Khusnutdinova reported on alkene hydrogenation utilizing a manganese(I) tricarbonyl complex featuring a picolylphosphine ligand.^32^

**Scheme 11:**
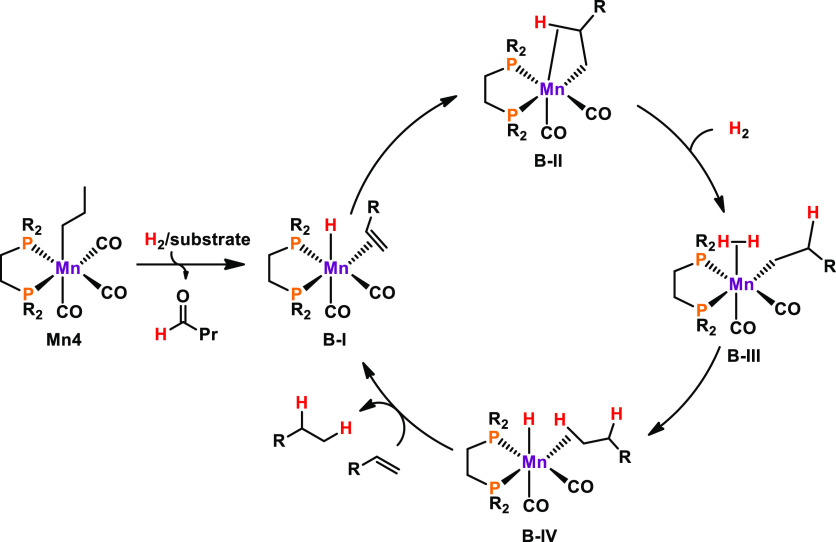
Simplified Catalytic Cycle of the Hydrogenation of Monosubstituted Alkenes

The reaction mechanism was studied by means of theoretical calculations (Scheme 11). Upon activation, **B-I** is formed. Hydride insertion gives complex **B-II**, which is stabilized by a C–H agostic interaction. Ligation of hydrogen gas gives rise to alkyl dihydrogen complex **B-III**, which is capable of splitting dihydrogen, thus yielding species **B-IV**. Finally, complex **B-I** is regenerated by the substitution of weakly C–H bonded alkane by the alkene substrate under product release.

**Scheme 12:**
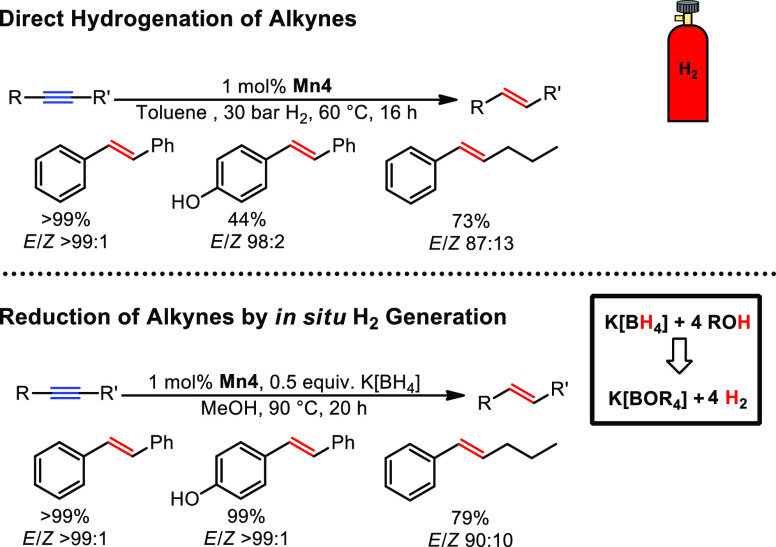
Semihydrogenation of Alkynes Catalyzed by **Mn4**

Encouraged by the high reactivity in the hydrogenation of C=C bonds, we decided to investigate the potential of manganese alkyl carbonyl compounds in alkyne reduction. Gratifyingly, **Mn4** showed high reactivity and selectivity in the semihydrogenation of disubstituted alkynes.^33^ In fact, remarkable *E*-selectivity was achieved with a catalyst loading of merely 1 mol % at 60 °C under 30 bar hydrogen pressure (Scheme 12). Furthermore, we envisioned semihydrogenation with in situ generated hydrogen gas and thus without the need of costly high-pressure setups, e.g., autoclaves. For this purpose, borohydrides in combination with alcohols as solvents were chosen as reagent mixture to provide hydrogen gas in situ. Fortunately, high reactivity and selectivity could be achieved at 90 °C. In addition to that, sensitive functional groups such as esters or acetals were left unaltered under the given reaction conditions.

**Scheme 13:**
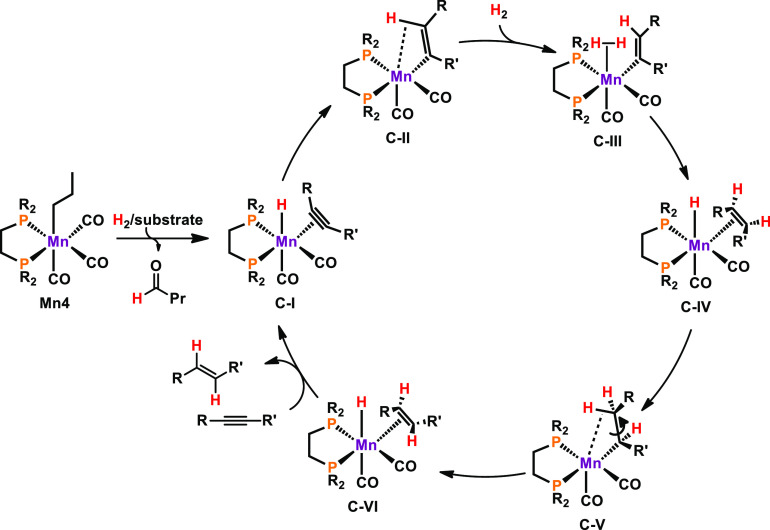
Simplified Reaction Mechanism for the Semihydrogenation of Alkynes

Mechanistic studies based on DFT calculations and accompanied by experimental findings were carried out. A simplified reaction mechanism is depicted in Scheme 13. Activation of **Mn4** leads to the formation of hydride species **C-I**, featuring an η^2^-coordinated alkyne ligand. Consecutive hydride attack gives rise to the unsaturated vinyl-ligated complex **C-II**, which binds hydrogen gas to yield **C-III**. Upon heterolytic cleavage of the H–H bond, hydride compound **C-IV** is formed, being ligated by an alkene in the *Z*-configuration. Hydride insertion delivers unsaturated species **C-V**, which undergoes β-hydride elimination to give **C-VI**. Within this complex, the alkene ligand holds an *E*-configuration. The catalytic cycle is closed by the coordination of fresh alkyne substrate upon product release. Recently, the groups of Beller^34^ and Rueping^35^ employed pincer-type manganese complexes for the *Z*-selective semihydrogenation of internal alkynes.

## Hydrofunctionalization Reactions

**Scheme 14:**
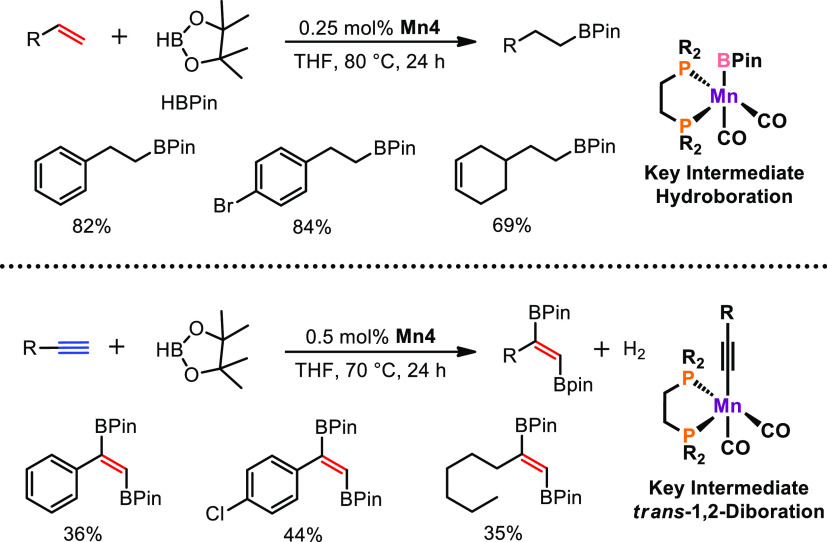
Efficient Hydroboration of Alkenes and *trans*-1,2-Diboration of Terminal Alkynes Catalyzed by **Mn4**

Motivated by the high reactivity in a broad variety of hydrogenation reactions, we wondered if manganese alkyl carbonyl complexes are also able to activate E–H bonds beyond dihydrogen. For this purpose, we decided to investigate the activation of hydrogen bonds in which the hydrogen atom is negatively polarized as it is in boranes or silanes. Remarkably, we observed high reactivity and selectivity in the anti-Markovnikov hydroboration of alkenes (Scheme 14).^2^ It should be noted that, thus far, only manganese complexes in the oxidation state of +II were utilized for hydroboration reactions of alkenes and alkynes by the groups of Zhang and Zheng,^36^ Thomas,^37^ Karton and de Ruiter,^38^ and Rueping.^39^ An array of terminal alkenes was efficiently hydroborated, tolerating a broad variety of functional groups such as halides, ethers, esters, and amines with a catalyst loading of merely 0.25 mol % **Mn4** for most substrates. Preliminary studies indicate that the key intermediate is an unsaturated boryl-ligated complex which is able to coordinate alkene substrate on the vacant side.

**Scheme 15:**
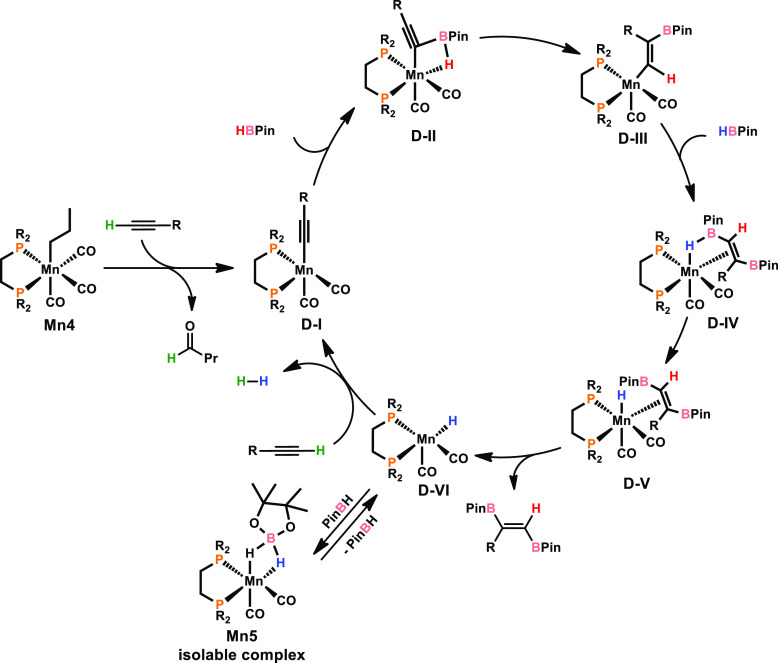
Simplified Reaction Mechanism for the *trans*-1,2-Diboration of Terminal Alkynes

Furthermore, **Mn4** is also capable of performing *trans*-1,2-diboration of terminal alkynes, which is accompanied by massive hydrogen production. It should be noted that **Mn4** is thus far the only transition metal complex which can catalyze this transformation. Detailed mechanistic studies were carried out to propose a reaction mechanism for the *trans*-1,2-diboration of terminal alkynes. For this purpose, deuterium-labeling experiments confirmed that the proton on the C=C bond has its origin in HBPin and not in the acetylene substrate. Furthermore, possible monoborated intermediates were independently synthesized but did not show any reactivity in the title reaction. Hence, a concerted reaction mechanism seems to take place. Thus, a reaction mechanism was formulated based on experimental data and extensive DFT calculation. A simplified catalytic cycle is shown in Scheme 15.

Within this context, **Mn4** is transformed to η^1^-ligated alkyne complex **D-I** upon C–H activation. Addition of pinacolborane (HBPin) to the unsaturated species results in the formation of compound **D-II**. Consecutive isomerization gives rise to the vinyl bonded complex **D-III**, which is able to coordinate a second equivalent of HBPin to yield **D-IV**. Breakage of the B–H bond gives hydride complex **D-V**, in which the product is weakly coordinated to the manganese center. Product release provides the unsaturated species **D-VI**, which reacts with fresh alkyne substrate to yield **D-I** under release of hydrogen gas. Alternatively, complex **D-VI** may also react with pinacolborane to yield **Mn5**, being stabilized by a κ^2^-ligated H_2_BPin ligand. In fact, **Mn5** represents an isolable compound, which was synthesized upon treatment of **Mn4** with HBpin. It is noteworthy that **Mn5** shows similar productivity in comparison to **Mn4** in *trans*-1,2-diboration of terminal alkynes as well as in hydroboration of alkenes. Thus, **Mn5** seems to be a dormant species in (hydro)boration reactions and can be reactivated upon release of neutral pinacolborane.

**Scheme 16:**
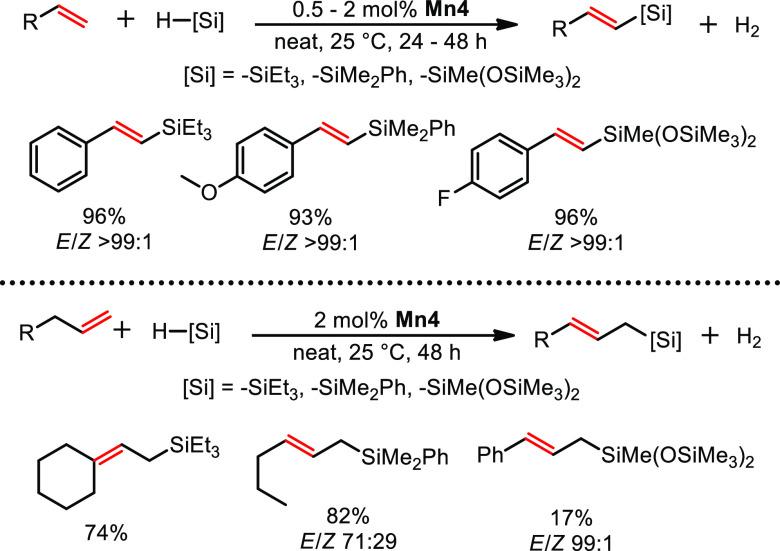
Dehydrogenative Silylation of Alkenes Yielding Vinyl- and Allyl Silanes Catalyzed by **Mn4**

Animated by the high reactivity in (hydro)boration reactions, we focused on the utilization of manganese alkyl carbonyl complexes in hydrosilylation of alkenes. However, conventional hydrosilylation of the C=C bond was not observed. In fact, reaction of alkenes with tertiary silanes exclusively gave dehydrogenative silylation (DS) products in high *E*-selectivity (Scheme 16).^3^ The reaction proceeds at room temperature without any solvent and catalyst loadings between 0.5 and 2 mol %, depending on the steric demand of employed silanes. Thus far, other manganese-based DS reactions operate at reaction conditions above 100 °C.^40^ In case of styrene derivatives vinyl silanes were formed whereas the reaction of aliphatic alkenes gave allyl silanes. This is likely attributed to γ-hydride elimination instead of β-hydride elimination.

**Scheme 17:**
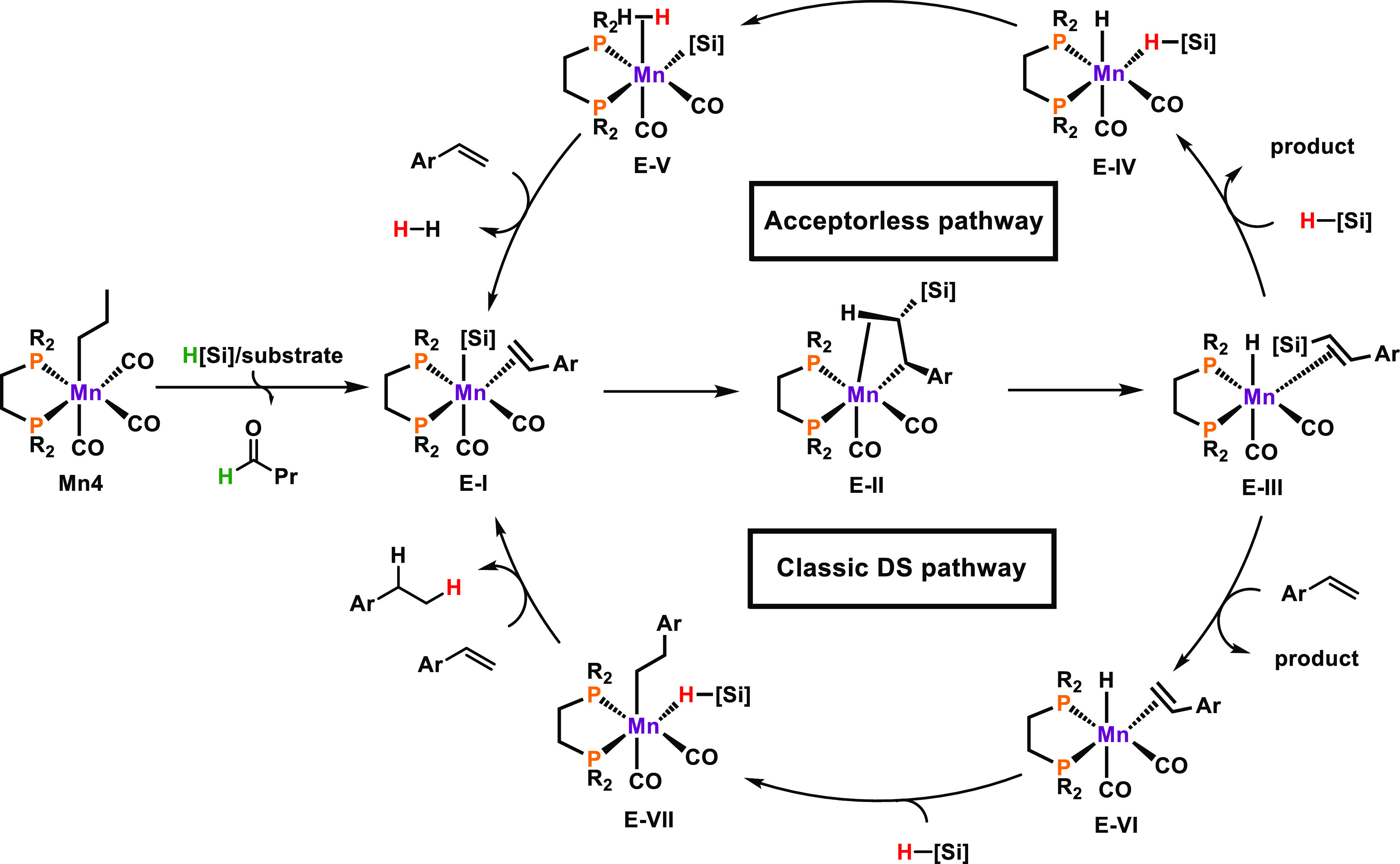
Simplified Reaction Mechanism for DS of Aromatic Alkenes Following Two Parallel Pathways

Usually, 1 equiv of alkene substrate is required to quench the in situ generated hydride complex in DS reactions. This results in the formation of one equiv of alkane as side product per equiv. DS-product.^41^ It should be noted that the ratio of DS-product to alkane approaches 3:2 rather than the usual 1:1 ratio in the presented DS reaction. Thus, mechanistic considerations were attempted to explain this finding. Experimental studies included kinetic data, deuterium labeling experiments, in operando NMR analysis, and structural determination of decomposed active species. Furthermore, headspace analysis revealed that hydrogen gas is formed during the reaction. In addition, DFT calculations provided further insight into the reaction mechanism for styrene derivatives, giving rise to two parallel pathways (Scheme 17). **Mn4** is activated upon migratory insertion, initiated by silane coordination, followed by substitution of formed *n*-butanal by alkene substrate to give silyl-bonded complex **E-I**. Nucleophilic attack of the silyl ligand results in the formation of **E-II**, being stabilized by a C–H agnostic interaction. Next, β-hydride elimination gives complex **E-III**. At this point, two reaction pathways may be followed to complete the catalytic cycle. The upper scenario represents the acceptorless pathway in which the coordinated product is released upon coordination of silane to **E-III** yielding hydride species **E-IV**, which features an η^1^-HsiR_3_ ligand. This compound may undergo the formation of **E-V**, which is able to release dihydrogen gas upon substitution with alkene substrate, thereby restoring **E-I**. Alternatively, **E-III** may also follow a “classic” pathway by substitution of the coordinated product by an alkene substrate (**E-VI**). Hydride attack followed by Si–H bond activation and consecutive coordination of fresh substrate then regenerates **E-I**. Experimental observations and theoretical calculations revealed that the acceptorless pathway dominates at low hydrogen partial pressure, i.e., at low conversions, whereas increased hydrogen content in the reaction mixture favors the route requiring a sacrificial alkene substrate.

## Carbon–Carbon Bond Forming Reactions

**Scheme 18:**
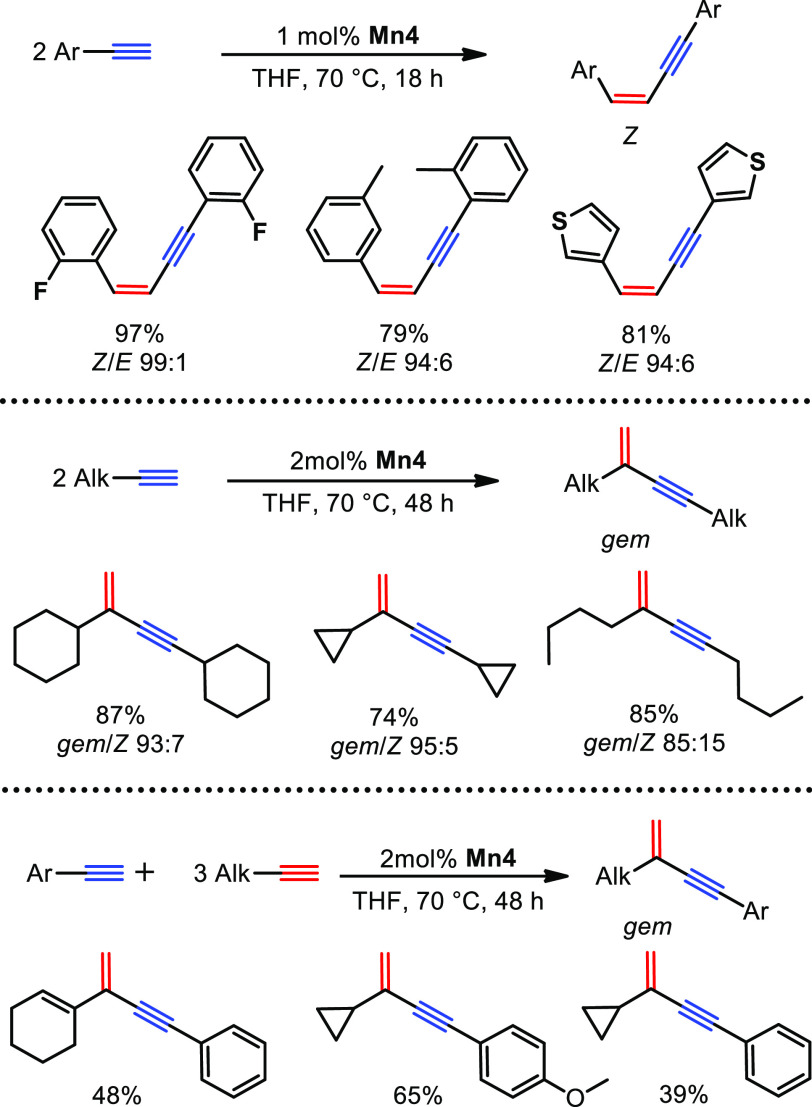
Dimerization and Cross-Coupling of Terminal Alkynes Catalyzed by **Mn4**

Furthermore, our group was interested in the activation of E–H bonds wherein the hydrogen atom possesses a positive polarization. For this purpose, we investigated the activation of the acidic C–H bond in terminal alkynes. In fact, **Mn4** was found to be an efficient catalyst for the dimerization of terminal alkynes (Scheme 18).^4^ In the case of aryl-substituted alkynes, high selectivity toward the head-to-head *Z*-1,3-enynes was found. Substrates bearing electron withdrawing groups showed the highest reactivity and selectivity. Aryl alkynes containing electron donating groups gave lower reactivity and/or selectivity. This is attributed to the decreased C–H acidity in electron-rich aryl substrates. Thus, C–H bond activation upon catalyst activation or within the reaction progress seems to be the limiting step within this catalytic transformation. This was also underlined by a kinetic isotope effect (KIE) of 1.49 for phenylacetylene vs phenylacetylene-*d*_1_.

Interestingly, the dimerization of alkyl-substituted alkynes resulted in the formation of large amounts of head-to-tail *gem*-1,3-enynes and minor content of expected head-to-head *Z*-1,3-enyne product. The lower acidity of the C–H bond in alkyl-based in comparison to aryl-based substrates results in lower reactivity for alkyl alkynes. A KIE of 2.44 was found for 1-octyne vs 1-octyne-*d*_1_. Furthermore, cross-dimerization of aryl- with alkyl-substituted alkynes, giving head-to-tail *gem*-1,3-enynes, could be achieved with **Mn4**. The highest selectivity was obtained for electron-rich aryl substrates in combination with alkyl alkynes. This is attributed to the lower tendency of electron-rich alkynes to undergo homocoupling instead of cross-coupling.

**Scheme 19:**
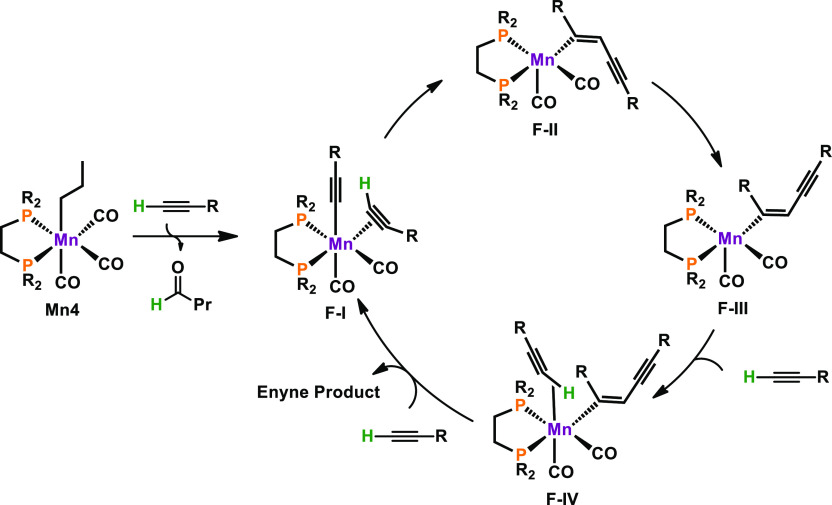
Simplified Catalytic Cycle for the Dimerization of Aromatic Alkynes

A plausible reaction mechanism was established based on DFT calculations as can be seen in Scheme 19. At first, C–H activation of the alkyne functionality followed by coordination of a second equivalent of substrate, being η^2^-bonded, leads to **F-I**. Attack of the η^1^-ligated alkyne donor on the C≡C bond yields vinyl coordinated complex **F-II**. In this intermediate, the 1,3-enyne holds an *E*-configuration. Double bond isomerization to the *Z*-isomer gives unsaturated species **F-III**. Coordination of a third equivalent of substrate results in the formation of **F-IV**. Finally, product release upon substitution with alkyne substrate closes the catalytic cycle. In the case of alkyl substrates giving head-to-tail *gem*-1,3-enynes, the η^2^-bonded alkyne ligand in **F-I** rotates 180° prior to nucleophilic attack of the anionic alkyne ligand.

## Summary and Outlook

Inspired by the migratory insertion of the alkyl group into carbon monoxide to yield coordinatively unsaturated complexes, our group reported on various applications of bisphosphine-supported manganese(I) complexes for the activation of moderately or nonpolarized bonds. In fact, the employed manganese alkyl carbonyl compounds were able to activate E–H (E = H, B, C, Si) bonds in a catalytic fashion. This results in the hydrogenation of polarized C–X (X = O, N) multiple bonds under base-free conditions. Furthermore, rare examples of manganese-catalyzed (semi)hydrogenation of alkenes and alkynes were presented. Due to rational design of steric and electronic parameters in the ligand set, high reactivity could be achieved under mild reaction conditions. Moreover, the developed manganese alkyl complexes are also capable of catalyzing dehydrogenative silylation of alkenes under partially acceptorless conditions. The reaction of alkenes with pinacolborane resulted in the selective formation of the anti-Markovnikov isomer, whereas terminal alkynes gave *trans*-1,2-diborated products under fully acceptorless conditions. In addition to hydrogenation and hydrofunctionalization reactions, the novel manganese alkyl carbonyl compounds can also be leveraged in carbon–carbon bond forming reactions, e.g., in the homo- and cross-dimerization of terminal alkynes.

Future applications may involve activation of N–H and O–H bonds in hydrofunctionalization of alkenes and alkynes or hydrogen production from suitable feedstocks. For this propose, the choice of mono-, bi-, and tridentate ligands, including mixed donor sets, may enhance the reactivity and stability of well-defined manganese complexes. In light of all the transformations thus far achieved, the potential of active Mn(I)-based systems opens up the way for conceptually and mechanistically well-founded research, which might lead to new developments and the discovery of novel catalysts extending the current scope and limitations of reactivity.
